# Harmful Effect of Beer on Bovine Enamel Microhardness – *In Vitro* Study

**DOI:** 10.1371/journal.pone.0163440

**Published:** 2016-10-19

**Authors:** Rayssa Ferreira Zanatta, Maria Ângela Lacerda Rangel Esper, Marcia Carneiro Valera, Renata Marques Melo, Eduardo Bresciani

**Affiliations:** 1 Department of Restorative Dentistry, Institute of Science and Technology, São Paulo State University—UNESP, São José dos Campos, São Paulo, Brazil; 2 Department of Dental Materials and Prosthodontics, Institute of Science and Technology, São Paulo State University—UNESP, São José dos Campos, São Paulo, Brazil; University of Brescia, ITALY

## Abstract

This study aimed to evaluate the effect of beers on the bovine enamel microhardness. Fifty rectangular (1 x 3 x 1 mm–height x width x thickness) enamel specimens were obtained from permanent bovine incisors, and divided into five groups (n = 10) according to the treatment employed: Saliva, Coke, Brahma, Heineken, and Budweiser. Microhardness (Knoop) were obtained before; after 5, 30 and 60 min of immersion in each solution. The data were analyzed using repeated two-way ANOVA and Tukey´s test (p<0.05). Coke decreased the microhardness in all immersion times, and Heineken, showed low values after 60 minutes. Beers tested have low potential to cause enamel erosion when compared to Coke.

## Introduction

Tooth erosion is a multifactorial process [1], mainly caused by patient’s diet, which involves the chemical removal of minerals from dental surface, initially from enamel followed by dentin [2]. It is defined as an irreversible loss of oral hard tissues due to a chemical dissolution promoted by intrinsic or extrinsic acids, and also chelating agents, without involvement of bacteria [3,4]. The extrinsic sources include the low pH beverages and food containing high concentration of acids [3]. Among the intrinsic causes of erosion there are the frequent regurgitation of stomach fluids, alimentary disorders, like bulimia and xerostomia or decrease in saliva buffering ability [5]. Alcoholism is also a factor that deserves consideration, as a previous study showed that 92% of alcoholics had erosive lesions [6].

The process starts with softening of enamel surface, mainly characterized by decrease in its microhardness [3]. Changes in dietary habits and increase in consumption of acid drinks and foodstuff has made erosion a growing concerning issue [7]. Tooth erosion usually occurs slowly and without pain, when restricted to enamel, and dental professionals should be aware of lesions first signs, inform the patients of the possible causes and stops the process as soon as possible [7].

Beer is the result of a complex fermentation series of starch products, mainly malted barley, wheat, rice and maize, for the lager type ones. Nowadays it is the third most commonly consumed beverage in the world [8]. In tropical countries, the beer consumption is about 57 liters/person/year [8]. There are few studies evaluating the erosive potential of beers, and usually they use analysis of calcium and phosphate release. Although beer had shown a lowest dissolving action compared with fruit juices and sports drinks [9], Nogueira et al.[10] evaluated some beers with pH from 3.79 to 4.80 using titratable acidity and concluded that they have potential erosive effects, as the critic pH for enamel dissolution is between 5.0 and 5.7 [11]. Nonetheless, to the best of our knowledge, studies evaluating the effect of beers over the enamel surface microhardness, characterizing the initial stages of erosion, are scarce.

The aim of this study was to evaluate the effect of lager beers on the bovine enamel microhardness, by means of laboratorial *in vitro* test. The work hypothesis is that beer does not decrease the surface microhardness of enamel.

## Materials and Methods

Sample size was calculated based on the mean of sound and demineralized enamel microhardness of a pilot experiment, considering α equals to 0.05 and power at 80%. From that, a minimum of 8 specimens per group was required. Fifty buccal surface specimens (n = 10) from extracted bovine permanent incisors were selected for the microhardness test. The bovine incisors were obtained from a slaughterhouse, after Ethics Committee approval (04/2015-CEUA-ICT-SJC-UNESP).

The specimens were cut, perpendicular to the buccal surface, with dimensions of 1x3x1 mm (height x width x depth) using a water-cooled diamond precision saw (Labcut 1010; Extec Technologies, Enfield, CT, USA). The specimens were embedded in acrylic resin (Jet; Classico, Sao Paulo, SP, Brazil), leaving the enamel surface uncovered, which were further polished flat (silicon carbide paper: grit 800–30 seconds, grit 1200 for 60 seconds and grit 4000 from 120 seconds), using a circular polishing machine, with speed of 300 rpm. During the exchange of grinding paper, the specimens were extensively washed with distilled water, and lastly they were ultrasound cleaned for 10 minutes with 1% NaOCl, followed by disinfection with 70% alcohol for 5 min [12]. Pilot investigation showed that 10 min immersion in NaOCl did not caused enamel demineralization.

Three brands of beer manufactured and commercialized in Brazil were tested (Table 1). They were analyzed with respect to acidity (pH), and change of enamel microhardness. negative and positive controls it was used artificial saliva (Kin Hidrat spray; Barcelona, Spain) and Coca-Cola (Porto Real, RJ, Brazil), respectively. All beers were lager type, bottled and used at environment temperature (around 25°C ± 2°C). The beers, artificial saliva and coke were acquired in local markets in the city of Sao Jose dos Campos, SP, Brazil.

**Table 1:** Description of the beers used.

Beer	Manufacturer	Composition
**Brahma (Bra)**	Anheuser–Busch InBev	Non-malt cereals, Water, Hop, Barley Malt, Yeast, Carbohydrates, antioxidant INS 316, INS 221 and stabilizing INS 405. Alcoholic contents: 4.8%
**Heineken (Hei)**	Heineken International.	Pure Barley Malt, Water, Hop, Yeast. Alcoholic contents: 5%
**Budweiser (Bud)**	Anheuser–Busch InBev	Non-malt cereals, Water, Hop, Barley Malt, Yeast. Alcoholic contents: 5%
**Coke (Cok)**	Coca-Cola Company	carbonated water, sugar, caramel colorant E150d, phosphoric acid as the acidifier, plant extracts and flavoring caffeine.
**Kin Hidrat (Sal)**	Kin	Potassium thiocyanate, potassium chloride, sodium chloride, calcium chloride, magnesium chloride, potassium dihydrogen phosphate, xylitol, saccharin sodium, hydrogenated castor oil PEG-40, Sodium Methylparaben, Sodium Propylparaben, Bonoprol, Menthol, Fragrance, Citric Acid, Water Purified.

Specimens were divided into five groups (n = 10) according to the erosive challenge employed: Sal (negative control group): specimens were immersed in artificial saliva; Cok (positive control group): specimens were immersed in Coke; Bra: specimens were immersed in Brahma beer; Hei: specimens were immersed in Heineken beer; Bud: specimens were immersed in Budweiser beer.

The pH of the beers was determined by using a digital pH meter (Digimed DM-20; Digicrom Analitica Ltda., Sao Paulo, Brazil), with a glass electrode, previously calibrated at pH 4.0 and 6.86 with standard buffer solutions. Five pH measures were taken in 100 mL of each beverage.

Microhardness measurements were performed with a Knoop hardness tester (FM-700; Future-Tech Corp., Tokyo, Japan). The measured parameter was the indentation length, often expressed in Knoop hardness numbers (KHN). The indenter was pressed perpendicular to the enamel surface (90°) with a 50 g load for 10 sec. Each indentation was repeated three times with a distance of 100 μm from each other. The specimens were immersed in the respectively solution of each group, at room temperature (25°C ± 2°C) with agitation of 80 RPM, for one hour. Four microhardness values were obtained during this period: KHN_0_ (before immersion); KHN_5_ (after 5 minutes immersed in solution)_;_ KHN_30_ (after 30 minutes immersed in solution) and KHN_60_ (after 60 minutes immersed in solution). The solutions of each group were replaced for new ones after each microhardness measurement. Data were analyzed using repeated two-way ANOVA and Tukey´s test (5%).

## Results

The results of repeated two-way ANOVA for microhardness evaluation revealed significant differences among groups (p = 0.0000; df = 12; F = 9.387), times (p = 0.0000; df = 3; F = 42.324) and solution (p = 0.0000; df = 4; F = 22.539). The results of Tukey´s test are shown in Table 2. Coke produced a significant decrease in microhardness at all immersion times. The beers did not show differences for microhardness in the same time tested, except for Heineken which had lower KHN values after 30 and 60 min. Artificial saliva also did not cause changes in KHN values. The pH values for all solution tested are also listed in Table 2.

**Table 2:** Means and standard deviation (SD) for microhardness values for different solutions and times tested.

	pH	KMH_0_	KMH_5_	KMH_30_	KMH_60_
**Saliva**	6.08	299.1 (±58.5) Aa	288.8 (±41.0) Aa	288.5 (±52.8) Aa	278,94(±85.8) Aa
**Coke**	2.36	328.11(±56.1) Aa	171.6 (±49.8) Bb	77.1 (±24.6) Bc	65.5 (±25.1) Bc
**Brahma**	4.34	315.2 (±24.9) Aa	241.4 (±64.1) ABa	246.4 (±39.9) Aa	254.7 (±55.1) Aa
**Heineken**	4.35	323.0. (±15.5) Aa	278.3 (±30.1) Aab	231.92(±53.9) Abc	196.8 (±56.2) Ac
**Budweiser**	4.26	297.3 (±77.6) Aa	295.0 (±88.6) Aa	271.8 (±66.5) Aa	261.8 (±59.1) Aa

Uppercase letters shows difference within columns. Lowercase letters show difference within lines.

The use of Coke and Artificial saliva as positive and negative control groups represents two extreme situations that might have masked the erosive effect of the beers. Therefore, another repeated two-way ANOVA was performed without these groups, to evaluate the effect among beers only. Beer factor presented no difference (p>0.31; df = 2; F = 1.222), however, for time (p<0.001; df = 3; F = 12,383) and beer-time interaction (p>0.023; df6; F = 2.596) there were significant differences. Still, Heineken had the lower value after 30 and 60 minutes of exposure.

## Discussion

The work hypothesis tested was accepted considering that the Heineken beer caused decrease of the enamel microhardness. Beer is a very common drink spread all over the world, easily available, and is the most consumed alcoholic drink in Brazil, with rate of 57 liter per capita/year [8]. The influence of alcohol in the oral health is much studied; however, there are few studies relating it with dental erosion [9,10,13].

The high consumption of alcoholic beverages and the problems associated with it, such as, gastric problems, reflux episodes, and vomiting may play a role in the erosive process [14]. As a result of this, some dental surfaces are more affected by erosion in alcoholic patients: palatine surfaces of maxillary teeth, occlusal surfaces of posterior teeth, and incisal edges of anterior teeth [15]. Some habits associated with drinks expand its deleterious effect on the enamel and can increase tooth erosion because they cause xerostomia, as smoking, drug use and hangover [16].

Some authors suggested that erosion after exposure to beverages was not related only to the pH of the beverage, considering that no relationship between pH of the beverages and erosion degree was found, so the pH itself cannot be used to predict the erosive potential [17]. As shown in Table 2, the pH of all beers was lower than 5.0 (lower than the critical dissolution enamel pH), however it was not able to reduce enamel microhardness at 5 and 30 min, except for Heineken, which caused significant reduce of enamel microhardness after 30 and 60 minutes of exposure.

There are two important factors that can affect the erosive potential of a solution: low degree of saturation in relation to hydroxyapatite and fluorapatite [15], and presence of citrate, a substance capable of chelating the calcium of the teeth [15]. There is lack of research relating beer to erosion, and usually they evaluate the erosive effect of beers considering the saturation of calcium and phosphate, without analyzing the effects over enamel surface. A previous study with bovine enamel showed no erosive effect for beer (pH of 4.18), and only products with a pH lower than 4.0 caused erosion [13]. Nogueira et al. (2000) evaluated the effect of difference beer brands in relation to titratable acidity, calcium and phosphate concentrations and pH measurements, and observed that the beers may have potential dental effects, indicating calcium loss from the enamel over time. Also Jager et al. (2008) evaluated the erosive potential of several beverages, including lager beers, and found that it presented lower tissue loss than Coke, however they discuss that the beverages composition is a more relevant subject.

According to the German Beer Purity Law, the only ingredients in beer composition should be water, malted barley and hops [8]. However, Brazilian legislation allows the replacement of part of malted barley by supplements, such as maize, barley, rice, wheat, rye and oats, since this replacement not exceed 50% [8]. During fermentation in brewing, yeast are placed in the mixture and free amino acids are absorbed by them, leaving the peptides and polypeptides containing glutamate and aspartate, plus citrate and other organic acids (such as lactate, succinate, pyruvate) excreted from yeast as the main buffer system in beer [18]. As discussed by Lussi and Jaeggi [15], citrate can be erosive due its ability to chelate with calcium from hydroxyapatite. So, if the citrate generated during fermentation was not completely consumed during the brewing process, the final beer can have an erosive property. Also, during fermentation, the yeast is responsible for changing the buffer ability of the beer, the consumption of bases and acids excretion, increasing the amount of free hydrogen ions in the mixture, being responsible for pH reduction, usually over 4.8 [19].

From the beers tested, Heineken was the only one to present decrease in enamel hardness after 30 and 60 min (Table 2). Interestingly, Heineken is the only beer with pure barley malt, without presenting non-malted cereals in its composition, as Brahma and Budweiser, which present rice and corn, among others. The relation between the absence of other kinds of cereal, such as rice and corn, and the amount of citrate produced during brewing was not found in literature, but it might be speculated that the ability of Heineken in decreasing enamel microhardness had a relation with the possible high amount of citrate in its composition. Future research might include analysis of titratable acidity and the fluids dynamic ever enamel.

Maltose is also found in beers [10] and is an important disaccharide commonly used for fermentation of oral microorganisms, producing acids. In addition, maltose can be transformed into glucose in beer fermentation with cariogenic effect [10]. Even though beer has cariogenic effect, it did not promote decrease in enamel microhardness, evidencing that calcium loss was not sufficient to promote an important reduction in the microhardness values.

Conversely, Coke has a higher potential to cause reduction in the enamel microhardness and this matter has been discussed by previous authors [20,21]. Coca-Cola was the only beverage that could be related to dental erosion due to its low pH and high amount of citric and phosphoric acids in its composition. Both acids are well known to cause erosion due calcium chelation, promoting hydroxyapatite dissolution, and having a high buffering ability, requiring more time and saliva to neutralize them. [11,12] The high prevalence of dental erosion and the frequency of consumption are plausible risk factors for the development of dental erosion [20]. Some preventive strategies are necessary for patients suffering from erosion, including dietary advice, stimulation of salivary flow, optimization of fluoride regimens, modification of erosive beverages, and adequate oral hygiene measures [22].

Therefore, the results of this study showed that the lower pH was not able to promote enough reduction in microhardness when compared to Coca-Cola. Heineken presented differences in microhardness also with 30 minutes’ exposure, when compared to the other beverages tested; indicating that this beer is, indeed, different from the others. The time of 60 min used in this study is not clinically relevant, but high exposure times can be used to simulate a cumulative erosive effect that can be measurable [23]. So, the finding in this study indicated that beer have a low ability in decrease enamel microhardness, once it took 30 minutes to Heineken to start causing a significant reduction, and is very unlikely that a person stays with beer contacting his/her teeth for this period of time. However, the possible cariogenic effect of beer should be better studied in future research.

The use of bovine teeth in dental research has being supported in literature due its similarity with human teeth in composition [24]. Being an *in vitro* study, some limitations should be considered regarding the results of this study: saliva and acquired pellicle are important biological factors that can influence and modulate the erosive process over enamel surface, due their buffer capacity and remineralization ability. The absence of an erosive cycle to intercalate saliva and beer immersion might have increased the real effect of beers over enamel surface, so, care has to be taken when extrapolating the results of this *in vitro* study to an *in vivo* situation.

Further studies can be done to evaluate the effect of saliva in the reduction of enamel microhardness caused by beers, as well as others beer brands can be tested, once their composition can be slightly different than the ones tested.

## Conclusion

Considering the limitations of this *in vitro* study, the following conclusion can be drawn: Coke promoted changes in enamel surface microhardness after 5 minutes of exposure, similarly to Heineken, but with increase of exposure Coke was more erosive that Heineken. A significant reduction in microhardness was found for Heineken after 30 minutes of exposure. Although this *in vitro* model does not reflect clinical situation, it presents some information about alteration caused by beers over enamel surface.
